# 1-(4-Amino-2-Hydroxyphenyl)Ethenone Suppresses *Agrobacterium tumefaciens* Virulence and Metabolism

**DOI:** 10.3389/fmicb.2020.584767

**Published:** 2020-11-12

**Authors:** Jin-Wei Zhou, Ai-Qun Jia, Xiao-Juan Tan, Hong Chen, Bing Sun, Tian-Zi Huang, Yu He, Pei-Li Li, En-Qi Liu

**Affiliations:** ^1^School of Food and Biology Engineering, Xuzhou University of Technology, Xuzhou, China; ^2^School of Life and Pharmaceutical Sciences, State Key Laboratory of Marine Resource Utilization in South China Sea, Hainan University, Haikou, China; ^3^Anhui Provincial Key Laboratory of Molecular Enzymology and Mechanism of Major Diseases, Anhui Normal University, Wuhu, China; ^4^Luoyang Key Laboratory of Organic Functional Molecules, College of Food and Drug, Luoyang Normal University, Luoyang, China; ^5^School of Environmental and Biological Engineering, Nanjing University of Science and Technology, Nanjing, China

**Keywords:** *Agrobacterium tumefaciens*, quorum sensing, 1-(4-amino-2-hydroxyphenyl)ethanone, metabolomics, pathogenicity

## Abstract

The impact of 1-(4-amino-2-hydroxyphenyl)ethanone (AHPE) from the metabolites of endophytic fungus *Phomopsis liquidambari* on quorum sensing (QS) of *Agrobacterium tumefaciens* was evaluated for the first time in this study. Exposure to AHPE at concentrations ranging from 12.5 to 50 μg/mL, the β-galactosidase activity, acyl-homoserine lactone level, swimming motility, chemotaxis, and flagella formation were significantly inhibited. qRT-PCR quantification combined with the docking analysis demonstrated that AHPE affected the QS system of *A. tumefaciens* by repressing the transcriptional levels of *traI* and *traR* rather than signal mimicry. ^1^H NMR-based metabolic analysis indicated that the metabolism of *A. tumefaciens* was notably disturbed with AHPE treatment. AHPE treatment also resulted in the enhanced oxidative stress in *A. tumefaciens*. The enhanced oxidative stress lead to the disorder of energy supply, protein synthesis, and nucleotide metabolism, and ultimately attenuated the pathogenicity of *A. tumefaciens*. Our study indicated that AHPE can serve as a potential pesticide to defend against *A. tumefaciens*.

## Introduction

*Agrobacterium tumefaciens* is a soil-borne phytopathogen that is responsible for crown gall disease in plant (Gelvin, 2003). It can affect more than 1000 various species of dicotyledonous plants and some monocotyledonous plants including many agronomically important crops (DeCleene and DeLey, 1976). *A. tumefaciens* causes crown gall disease by transferring and integrating its T-DNA into the plant genome and then inducing the biosynthesis of auxin and cytokinin (Gohlke and Deeken, 2014). Increased auxin and cytokinin levels result in uncontrolled development of crown galls due to the changed balance between these phytohormones. Crown gall disease caused by *A. tumefaciens* leads to substantial economic losses worldwide, mostly pertaining to perennial horticulture plants, by causing reduced growth, vigor, and yield, and severe diseases may cause partial or complete death of infected plants. An effective way to control crown gall disease is biological control by *Agrobacterium radiobacter* strain K84, a bacterium that can excrete agrocin 84 that is toxic to *A. tumefaciens* (Moore and Warren, 1979). However, this protection is limited because it is not suitable for all *A. tumefaciens* strains. Alternative measures depending on RNA interference to repress the expressions of genes involved in auxin synthesis were advocated for crown gall controlling (Lee et al., 2003). However, the methods were also limited for practical application due to the use of transgenic techniques. Therefore, developing durable disease control approaches for combating *A. tumefaciens* disease is needed.

T-DNA also encodes for synthesis of opines (Schell et al., 1979), which are specific growth substrates and signals used by the virulent *Agrobacterium* spp. Some opines, called conjugative opines, are required for synthesis of a quorum sensing (QS) signal, acylated homoserine lactones (AHLs) 3-oxo-octanoyl-homoserine (3-oxo-C8-HSL). The QS signal 3-oxo-C8-HSL was synthesized by the TraI AHL synthase (Zhang et al., 1993). The TraR protein is an AHL-responsive LuxR-type transcription factor that activates genes controlling Ti plasmid replication (*rep*) and conjugal transfer (Chen et al., 2004). At inducing concentrations, the AHL is bound by TraR, causing the formation of a stable, active homodimer with one AHL bound per TraR protomer. Dimerized TraR-AHL has an increased affinity for the control (*tra*-box) sequences upstream of target *rep* and *tra* operons, resulting in binding of the DNA and, in turn, transcriptional activation (Fuqua et al., 1996; Zhu and Winans, 1999; Chen et al., 2004). A link between a high copy number of Ti plasmid and severity of tumor symptoms was established (Pappas and Winans, 2003) and, consequently, anti-virulence strategies targeting QS have been proposed to attenuate *Agrobacterium*-induced symptoms on plants (Molina et al., 2003; Chevrot et al., 2006).

The chemical 1-(4-amino-2-hydroxyphenyl)ethanone (AHPE) (Supplementary Figure 1) was firstly purified from the metabolites of *Phomopsis liquidambari* S47, one endophytic fungus isolated from the leaves of *Punica granatum*. To date, literature about the effect of AHPE on QS and virulence of *A. tumefaciens* has not been reported. In this research, the impact of AHPE on QS was evaluated by using the reporter strain *A. tumefaciens* A136 (pCF218/PCF372) (*traI*: *lacZ*) (McLean et al., 1997) and the metabolic response of *A. tumefaciens* to AHPE was determined by using the ^1^H NMR-based global metabolomics.

## Materials and Methods

### Test Strains and Chemicals

*Phomopsis liquidambari* S47 was preserved in China Center for Type Culture Collection with the ID of M2018476. The AHL-responsive reporter strain *A. tumefaciens* A136 was kindly donated by Prof. Jun Zhu from University of Pennsylvania. The wild-type strain *A. tumefaciens* C58 was provided by Prof. Min-Liang Guo from Yangzhou University. All strains were cultivated in *A. tumefaciens* (AT) medium (K_2_HPO_4_ 10.5 g, KH_2_PO_4_ 4.5 g, MgSO_4_ 0.2 g, FeSO_4_ 5 mg, CaCl_2_ 10 mg, MnCl_2_ 2 mg, (NH_4_)_2_SO_4_ 2 g, mannitol 2 g, H_2_O 1000 mL, pH 7.0) at 28°C unless otherwise specified. The chemical 1-(4-amino-2-hydroxyphenyl)ethanone (AHPE) was purified from the metabolites of *P. liquidambari* S47 by forward and reversed silica gel chromatography, HPLC, and gel permeation chromatography.

### Growth Measurement

The minimum inhibitory concentration (MIC) of AHPE was determined according to the Clinical and Laboratory Standards Institute (Clinical and Laboratory Standards Institute (CLSI), 2011) with a bacterial concentration of 1–5 × 10^5^ CFU/mL. AHPE was serially two-fold diluted in AT medium. For growth profile analysis, overnight cultures of *A. tumefaciens* C58 was inoculated into 30 mL of AT medium to obtain OD_6__2__0_ = 0.05. The cultures were added with various concentrations of AHPE and then cultured at 28°C for 24 h. Salicylic acid (15 μg/mL) and DMSO served as the positive and negative control, respectively. Growth was analyzed by reading OD value at 620 nm (Biotek Elx800, United States).

### β-Galactosidase Activity

Overnight cultures of *A. tumefaciens* A136 (OD_6__2__0_ = 0.5) were 0.1% inoculated into 100 mL of AT medium with or without AHPE. Aliquots (300 μL) of 3-oxo-C8-HSL (1 mM, Sigma-Aldrich, United States) were added into the cultures and then incubated at 28°C for 17 h. Salicylic acid (15 μg/mL) and DMSO were used as positive and negative control, respectively. After incubation, the OD_6__0__0_ was determined. And then, 200 μL of cultures were added with 800 μL of Z-buffer, 100 μL of 0.1% SDS, 150 μL chloroform, and 100 μL of ONPG (4 mg/mL). The mixture was incubated at water bath at 28°C until the mixture turned yellow, and then 600 μL of 1 M sodium carbonate solution was added for reaction termination. The reaction time (T, min) was recorded. After centrifugation, the OD_420_ value was determined. The β-galactosidase activity was determined using the following formula as described previously (Stachel et al., 1985):

(1) $$\frac{{\text{A}}_{420}\times 1000}{{\text{A}}_{600}\times \text{T}\times 0.2\text{mL}}$$

### AHL Analysis

The potential impact of AHPE on QS was evaluated by measuring 3-oxo-C8-HSL level produced by *A. tumefaciens* C58 (Morin et al., 2003). Briefly, 50 μL cultures of *A. tumefaciens* C58 were inoculated into 50 mL of AT medium and then incubated with AHPE (12.5, 25, and 50 μg/mL) at 28°C for 17 h. DMSO served as the negative control. After centrifugation at 12000 rpm, the supernatant was extracted with the same volume of acidified ethyl acetate. The solvent was evaporated and residues were redissolved with methanol. The level of 3-oxo-C8-HSL was quantified by liquid chromatography-tandem mass spectrometry (LC-MS/MS) (Zhou et al., 2018b).

### Swimming and Chemotaxis

The swimming motility assay was performed according to Merritt (Merritt et al., 2007) with minor modifications. Briefly, 2 μL overnight cultures of *A. tumefaciens* C58 (OD_6__2__0_ = 0.5) were inoculated in the ATGN medium (K_2_HPO_4_ 10.5 g, KH_2_PO_4_ 4.5 g, MgSO_4_ 0.2 g, FeSO_4_ 5 mg, CaCl_2_ 10 mg, MnCl_2_ 2 mg, (NH_4_)_2_SO_4_ 15 mM, glucose 5 g, agar 3 g, H_2_O 1000 mL, pH 7.0) supplemented with various concentrations of AHPE (25 and 50 μg/mL). Salicylic acid (15 μg/mL) and DMSO served as the positive and negative control, respectively. After 24-h incubation, the swimming diameter was recorded.

The chemotaxis assay was evaluated as described by Ding (Ding and Christie, 2003). Briefly, 2 μL overnight cultures of *A. tumefaciens* C58 (OD_6__2__0_ = 0.5) were inoculated in one side of the ATGN medium (without glucose) supplemented with various concentrations of AHPE (25 and 50 μg/mL). One strip filter paper with glucose was placed 4 cm away from the bacterial solution and then incubated at 28^o^C for 24 h. Salicylic acid (15 μg/mL) and DMSO served as the positive and negative control, respectively. After incubation, the chemotaxis diameter was determined.

### Effect of AHPE on Flagella

Overnight cultures of *A. tumefaciens* C58 (OD_620_ = 0.5) were 0.1% inoculated into fresh AT medium supplemented with various concentrations of AHPE (25 and 50 μg/mL) and incubated at 28°C for 17 h. Salicylic acid (15 μg/mL) and DMSO served as the positive and negative control, respectively. After incubation, 1 mL of bacterial cultures were mixed with 50 μL of 37% methanol and then centrifugated at 5000 rpm for 2 min. Cells were washed with distilled water and then resuspended with 200 μL of distilled water. The resuspension (5 μL) was dropped on a slide, dried naturally, and then stained with the flagella staining solution (Beijing Solarbio Science and Technology Co., Ltd., China) for 5 min. After staining, the excess dye was rinsed off and flagella were observed under light microscope (Nikon 80i, Japan).

### Molecular Docking

Docking simulation was performed using the AutoDock 4.2 program. The structures of AHPE and 3-oxo-C8-HSL were drew with ChemBioDraw 12.0. All water molecules were removed in the protein files (TraR, PDB: 1H0M), polar hydrogen atoms were added, and non-polar hydrogen atoms were merged using the Hydrogen module in the AutoDock Tools (ADT). Gasteiger charges were assigned and the Lamarckian genetic algorithm was used to obtain the conformation of compounds in the binding pocket. The RMSD difference within 2 Å to give the best scored poses. Protein ligand interactions were obtained by the online tool PLIP^1^ and analyzed using PyMOL. A docking grid with a size of 50^∗^50^∗^50 was used. For each PDB structure, the center coordinate was obtained from active site. The default settings for docking in AutoDock 4.2 software were used except for num_modes (50). ADT shows the docking scores as affinity of binding, and the scores were expressed as kilocalories per mole (Zhou et al., 2018a).

### ^1^H NMR-Based Metabolic Analysis

*Agrobacterium tumefaciens* C58 was cultivated as described above with DMSO served as the negative control. Cells were harvested by centrifugation at 4°C and 12000 rpm, and then washed with precooled PBS. Cell pellets were added with methanol/H_2_O (3.8 mL, 1/0.9) and homogenized by 4-min intermittent sonication. After homogenization, 4 mL of chloroform was supplemented. The mixtures were centrifugated and the upper layer was collected. Methanol was removed with an organomation and then lyophilized. The dried metabolites were resolved in D_2_O and 0.05% (w/v) sodium 3-(trimethylsilyl) propionate-2,2,3,3-d4 (TSP) for referencing purposes. ^1^H NMR analysis and data processing were performed according to Chen (Chen et al., 2017). The fold changes in metabolites and associated *p*-values were presented in fold change plots.

### Gene Expressions

*Agrobacterium tumefaciens* C58 was cultivated as described above with DMSO served as the negative control. Cells were harvested by centrifugation at 4°C and 12000 rpm, and then washed with precooled sterile PBS. RNA was extracted using RNA extraction kit (Tiangen Biotech, Beijing, China) and the quantitative real-time PCR (qRT-PCR) reaction was performed according to Zhou (Zhou et al., 2019) with the reference gene *lepA* (*atu0241*) set as the internal control (Lang et al., 2016). The primers were listed in Supplementary Table 1.

### Tobacco Infection Assay

Tobacco infection assay was performed as described by Liu (Liu et al., 2012) and Dandurishvili (Dandurishvili et al., 2011). Briefly, *A. tumefaciens* C58 was 0.1% inoculated into AT medium containing 25 and 50 μg/mL of AHPE, and incubation at 28°C and 180 rpm for 17 h. Salicylic acid (15 μg/mL) and DMSO were used as the positive and negative control, respectively. Cells were harvested by centrifugation, resuspended in PBS buffer to obtain an OD_6__2__0_ = 0.1 (approximately 1 × 10^8^ cfu/mL). Stems of four-week-old tobaccos were inoculated by slight injury to the stem using a needle dipped in *A. tumefaciens* C58 suspension. The blank control was sprayed with water. Pictures and tumor weight were recorded after four-week of infection.

## Results

### MIC and Growth Profile

**FIGURE 1:**
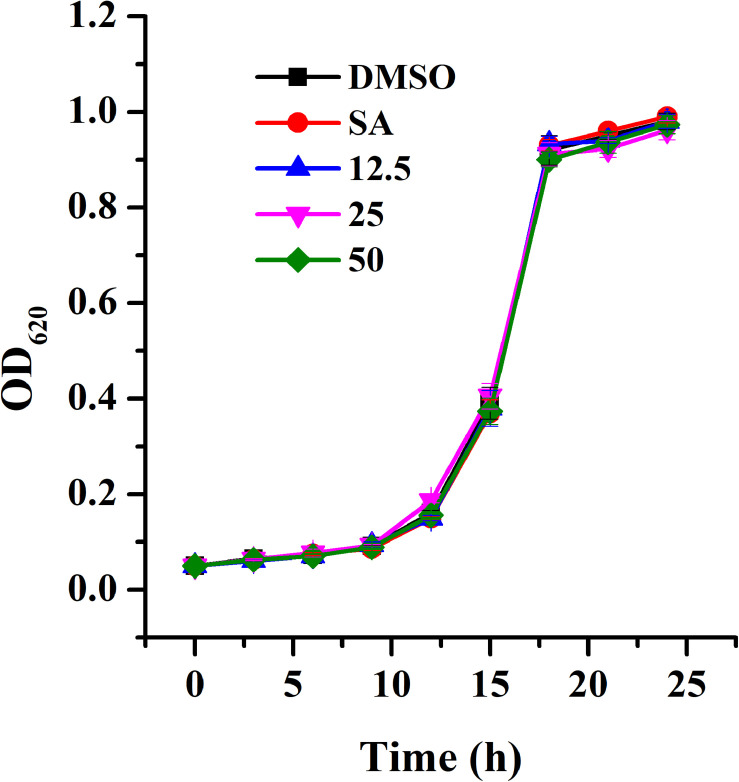
Growth profile of *A. tumefaciens* treated with AHPE. Growth was evaluated at various concentration of AHPE (12.5, 25, and 50 μg/mL) for 24 h. DMSO and salicylic acid (15 μg/mL) were used as the negative and positive controls, respectively. Error bars represent standard deviations of three measurements.

The MIC of AHPE for *A. tumefaciens* strains was determined to be 100 μg/mL. The growth analysis was evaluated by employing AHPE at sub-MIC concentrations. Results indicated that exposure to AHPE at 12.5, 25, and 50 μg/mL exhibited no inhibitory impact on bacterial growth (Figure 1).

### β-Galactosidase Activity

**FIGURE 2:**
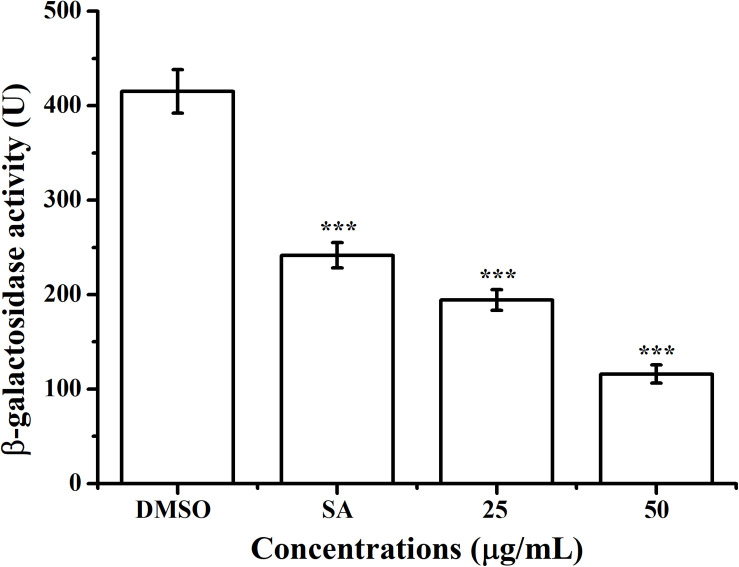
Effect of AHPE (25 and 50 μg/mL) on β-galactosidase activity using *A. tumefaciens* A136 as the AHL-responsive reporter strain. Salicylic acid (15 μg/mL) and DMSO were used as positive and negative control, respectively. Statistical differences were determined by ANOVA followed by Tukey-Kramer test. ***p < 0.001 versus the DMSO control.

The effect of AHPE on QS system of *A. tumefaciens* was evaluated by β-galactosidase assay by using the AHL-responsive reporter strain *A. tumefaciens* A136 which contained a *traI*:*lacZ* fusion (McLean et al., 1997). Exposure to AHPE at 25 and 50 μg/mL resulted in approximately 53 and 72% inhibition in β-galactosidase activity when compared with the DMSO control (Figure 2), which was more efficient than that of 15 μg/mL of salicylic acid (Figure 2).

### Evaluation of AHL Level

**FIGURE 3:**
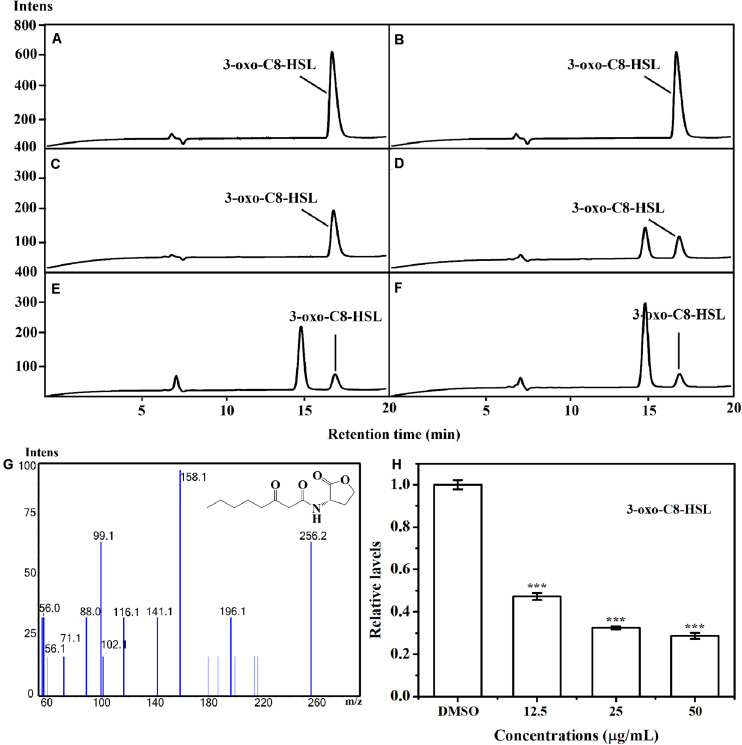
Relative quantification of 3-oxo-C8-HSL by LC-MS/MS chromatograms. HPLC chromatograms of 3-oxo-C8-HSL produced by *A. tumefaciens* C58 treated with **(C)** DMSO and **(D–F)** AHPE (12.5, 25, and 50 μg/mL, respectively). **(A)** and **(B)** represented the standard chemicals of 3-oxo-C8-HSL. **(G)** MS/MS spectra of 3-oxo-C8-HSL. **(H)** Quantitative analysis of 3-oxo-C8-HSL treated with 12.5, 25, and 50 μg/mL of AHPE, respectively. Statistical differences were determined by ANOVA followed by Tukey-Kramer test. ***p < 0.001 versus the DMSO control.

The putative impact of AHPE on QS of *A. tumefaciens* C58 was further evaluated by investigating the AHL level secreted by this organism. LS-MS/MS confirmed the presence of the signal molecule 3-oxo-C8-HSL in the supernatant of *A. tumefaciens* C58 (Figures 3C–G). Exposure to AHPE at 12.5, 25, and 50 μg/mL significantly decreased the peaks and areas of 3-oxo-C8-HSL, which was evidenced by the HPLC chromatograms (Figures 3D–F). Relative quantification indicated that AHPE treatment resulted in the reduced production of 3-oxo-C8-HSL by about 50, 65, and 70%, respectively, related to the DMSO-treated control (Figure 3H).

### Interference of Motilities and Flagella Formation

**FIGURE 4:**
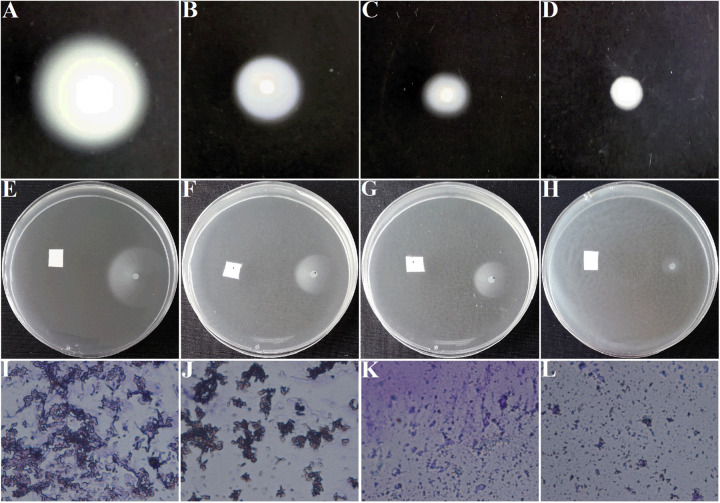
Effect of AHPE on swimming motility **(A–D)**, chemotaxis **(E–H)**, and flagella formation **(I–L)**. Images of **(A,E,I)** represented DMSO-treated groups; **(B,F,J)** represented 15 μg/mL of salicylic acid-treated groups; **(C,G,K)** represented 25 μg/mL of AHPE-treated groups; and **(D,H,L)** represented 50 μg/mL of AHPE-treated groups.

Motilities and chemotaxis are important indexes to evaluate the survival and infection abilities of *A. tumefaciens*. Excellent motilities and chemotaxis are beneficial for bacteria to strive for more nutrients and host infection in the environment (Erhardt, 2016). In addition, *A. tumefaciens* moves forward for survival and infection was governed by clockwise rotation of flagella (Guo et al., 2017). Therefore, inhibition of motilities, chemotaxis, and flagella are essential for suppressing the pathogenicity of *A. tumefaciens*. For the DMSO-treated group, the swimming diameter was about 6.5 ± 0.3 cm (Figure 4A). However, the swimming diameter was reduced to 2.0 ± 0.3 cm (Figure 4C) and 0.7 ± 0.1 cm (Figure 4D), respectively, when exposed to 25 and 50 μg/mL of AHPE. The inhibitory efficiency of AHPE was superior to that of salicylic acid (Figure 4B). AHPE also showed dose-dependently suppressed effect on chemotaxis, which was evidenced by the chemotaxis diameter (Figures 4E–H). After AHPE treatment (25 and 50 μg/mL), the diameter of chemotaxis was obviously decreased from 3.0 ± 0.2 cm (Figure 4E) to 1.4 ± 0.2 cm (Figure 4G) and 0.4 ± 0.2 cm (Figure 4H), respectively. Similarly, a notable suppression on flagella formation was also detected after treatment with AHPE (Figures 4K,L) when compared with the DMSO-treated control (Figure 4I). The inhibited efficiency of AHPE was superior to that of salicylic acid (Figure 4J).

### Docking Analysis

**FIGURE 5:**
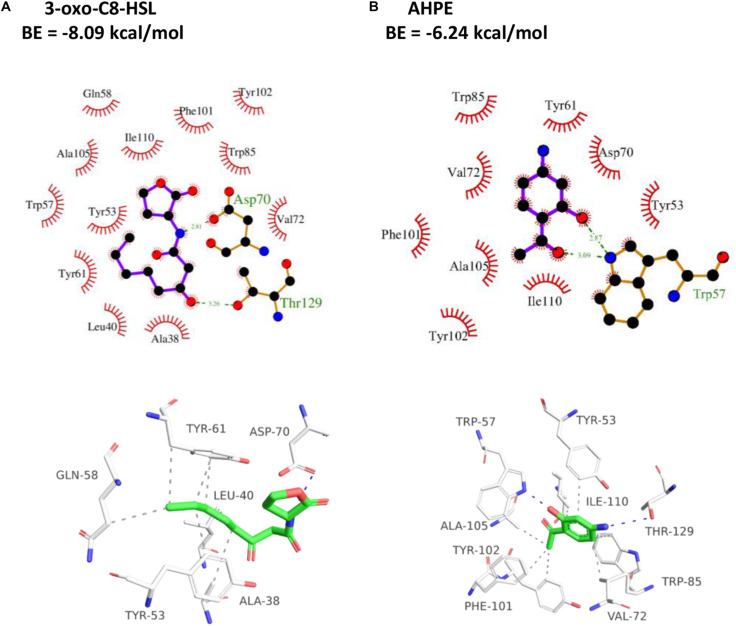
2D (top) and 3D (bottom) schematics of receptor-ligand interactions of TraR with 3-oxo-C8-HSL **(A)** and AHPE **(B)**, respectively.

As presented in Figure 5A, the amino residue of 3-oxo-C8-HSL showed H-binding interaction with Asp70 at a distance of 2.81 Å and the carbonyl group demonstrated H-binding interaction with Thr129 at a distance of 3.26 Å. The hydroxyl group and carbonyl group of AHPE showed H-interactions with Trp57, respectively (Figure 5B). AHPE binds to TraR with the energy of −6.24 kcal/mol, whereas 3-oxo-C8-HSL was −8.09, indicating that 3-oxo-C8-HSL has stronger binding affinity to TraR compared with AHPE.

### Metabolic Analysis Exposed to AHPE

**FIGURE 6:**
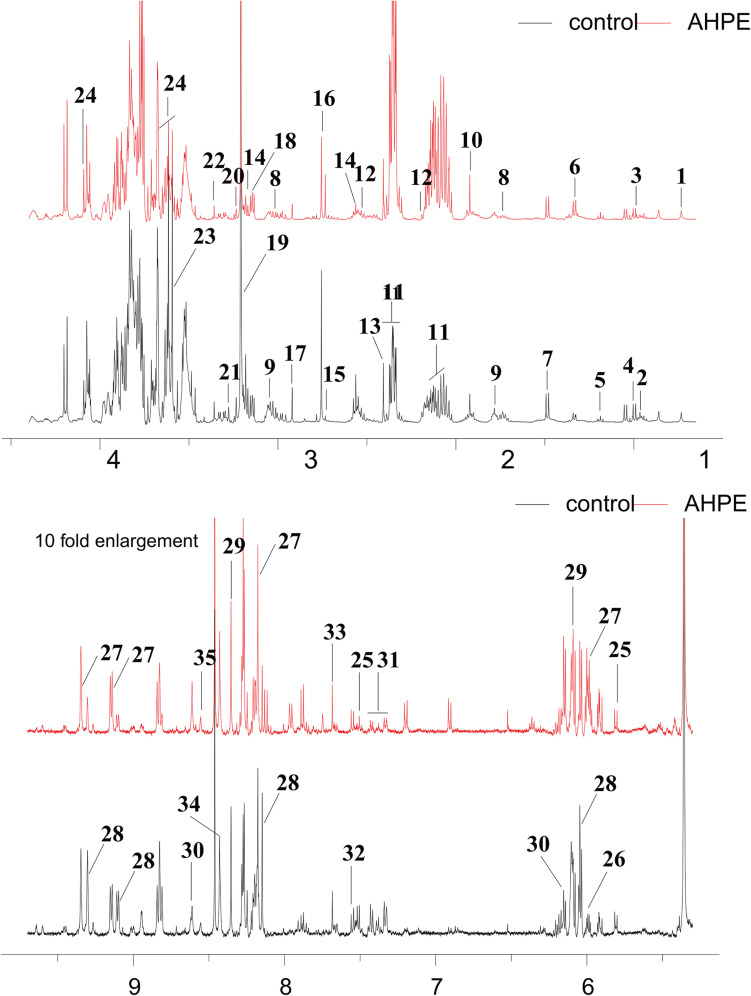
^1^H NMR spectra of *A. tumefaciens* extracts from AHPE-treated (red line) and control group (black line). Labeled metabolites were assigned in Table 1.

**FIGURE 7:**
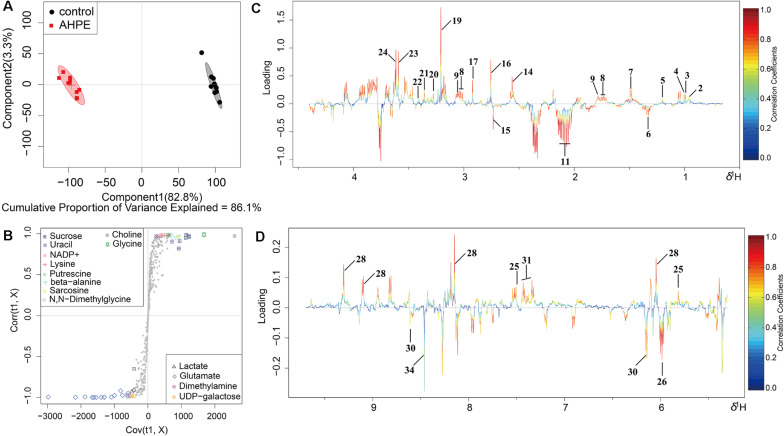
OSC-PLS-DA of metabolomics profiles from AHPE-treated and control groups. **(A)** PCA score plot. **(B)** S-plot points represent different variables (metabolites). **(C,D)** Color-coded loading plot after removal of water signals and affected regions.

Metabolic changes of *A. tumefaciens* C58 treated with AHPE were presented in Figure 6. A total of 35 metabolites including organic acid, organic amine, amino acids, and energy-related compounds were assigned according to their hydrogen chemical shift and peaks’ shape (Table 1). The PCA score plot showed a significant separation between the untreated and AHPE-treated groups (Figure 7A), which indicated that AHPE has a marked impact on metabolism in *A. tumefaciens* C58. From the S-plot (Figure 7B) and loading plots (Figures 7C,D), the levels of lysine, putrescine, beta-alanine, sarcosine, *N*, *N*-dimethylglycine, choline, glycine, sucrose, uracil, and NADP^+^ were significantly decreased, while lactate, glutamate, dimethylamine, and UDP-galactose were significantly increased after exposure to AHPE. The assignments and fold changes between groups were displayed in Table 1.

**TABLE 1:**
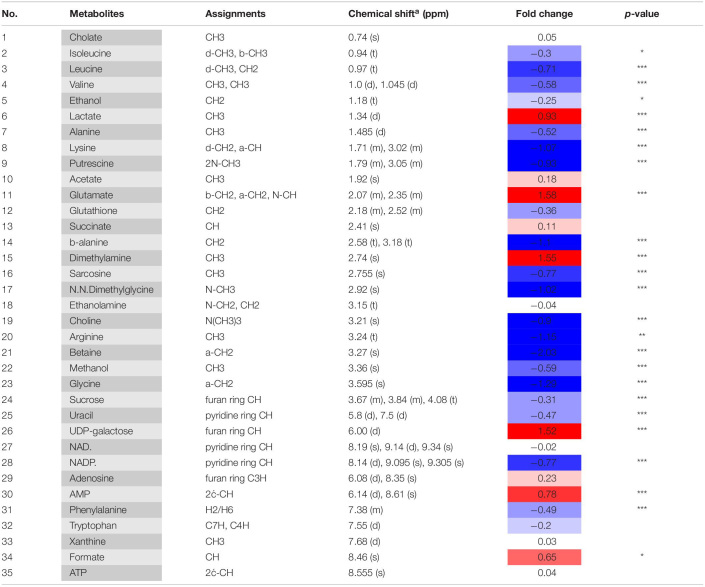
Metabolites in *A. tumefaciens* C58 identified by ^1^H NMR after dosing AHPE.

*Red and blue represented the increased and decreased metabolites, respectively, in AHPE-treated group.*

### QS and Virulence-Related Gene Expression

**FIGURE 8:**
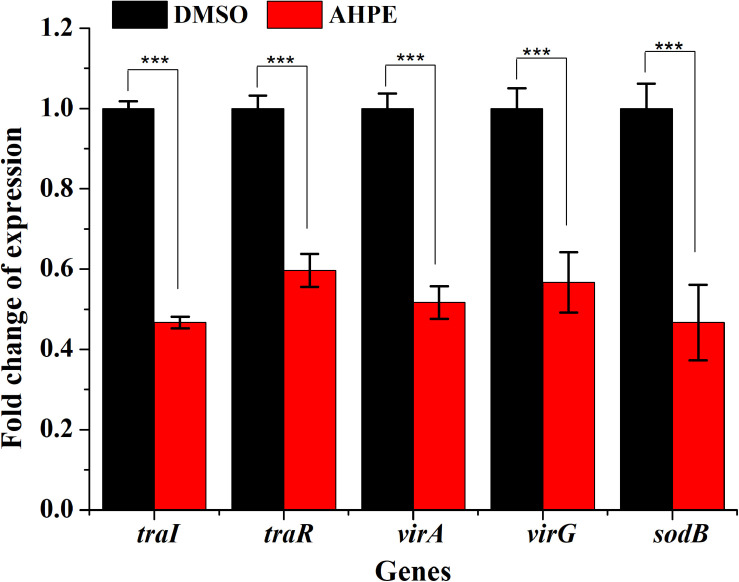
Effect of AHPE on expressions of genes involved in QS, virulence and antioxidant enzyme. ***p < 0.001 versus the DMSO control.

qRT-PCR was employed to evaluate the effect of AHPE on transcriptional levels of five genes involved in QS, virulence, and antioxidase, that is *traR*, *traI*, *virA*, *virG*, and *sodB*. Results indicated that AHPE exposure lead to a significant downregulation in the expressions of *traR* and *traI* by about 53 and 40%, respectively. Similarly, the expressions of *virA*, *virG*, and *sodB*, three genes involved in the synthesis of virulence factors and superoxide dismutase, were also notably reduced after exposure to AHPE (Figure 8). Therefore, we speculated that the downregulated expressions of these QS and virulence-related genes would inevitably lead to the reduced pathogenicity of *A. tumefaciens* C58.

### Tobacco Stem Infection Assay

**FIGURE 9:**
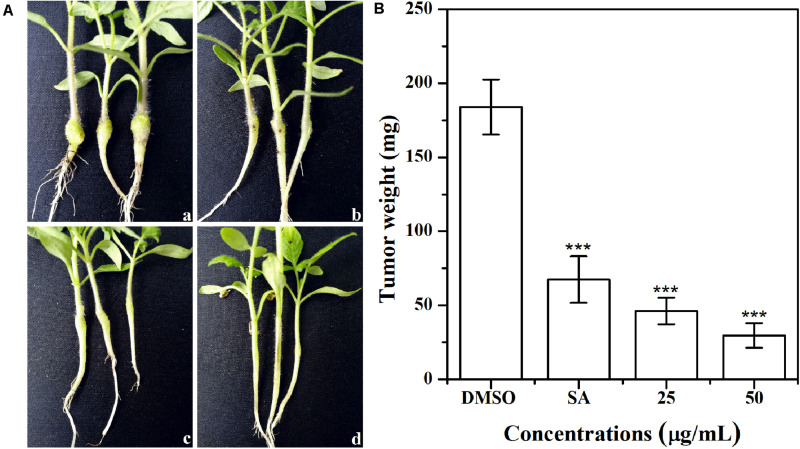
Effect of AHPE on tobacco plant infections. **(A)** Inoculation with *A. tumefaciens* C58 treated with **(a)** DMSO, **(b)** salicylic acid (15 μg/mL), **(c)** 25 μg/mL of AHPE, and **(d)** 50 μg/mL of AHPE, respectively. **(B)** Quantification of crown gall weight treated with or without AHPE. ***p < 0.001 versus DMSO or resveratrol-treated control.

The effect of AHPE on tobacco stem infection was investigated. As shown in Figure 9A, exposure to AHPE at 25 and 50 μg/mL significantly inhibited the formation of crown galls caused by *A. tumefaciens* C58. Quantification analysis indicated that the weight of crown galls was remarkably decreased after treatment with AHPE compared to the salicylic acid and DMSO-treated groups (Figure 9B). The result indicated that AHPE has the potential to function as a pesticide in preventing *A. tumefaciens* from infecting tobacco plants at seedling stage.

## Discussion

*Agrobacterium tumefaciens* is a soil-born phytopathogen that can cause crown gall disease in more than 1000 species of dicots and certain monocots. Crown gall disease sometimes will result in significant economic losses by bringing disastrous crop failure. During the infection, T-DNA is transferred from *A. tumefaciens* plasmid into plants and integrated into plant genome (Hao et al., 2007). And then, the integrated genes induce the synthesis of auxin and cytokinin, disrupt the balance of plant hormones, lead to the formation of tumor, and ultimately result in the death of plants. Studies have shown that conjugal transfer (*tra*) genes were mediated by QS (Fuqua and Winans, 1994). Therefore, interfering with the QS would be an efficient strategy for attenuating the virulence of *A. tumefaciens*. In this study, a natural compound, 1-(4-amino-2-hydroxyphenyl)ethanone (AHPE), was firstly isolated from the metabolites of *P. liquidambari* S47. However, the anti-QS and anti-virulence activities of AHPE against *A. tumefaciens* have not been documented. Here, AHPE was evaluated for its potential to inhibit the QS and relative virulence of *A. tumefaciens*. AHPE showed a concentration-dependent reduction in β-galactosidase activity and AHL production, thus indicating the breakdown of QS in *A. tumefaciens*. AHPE also disturbed the metabolic profiles of *A. tumefaciens* as evidenced by the altered metabolites involved in oxidative stress, energy metabolism, protein synthesis and nucleotide metabolism.

The effect of AHPE on QS was firstly evaluated by determining β-galactosidase activity using *A. tumefaciens* A136. Results showed significantly reduced β-galactosidase activity after treatment with AHPE, thus indicating the breakdown of QS in *A. tumefaciens*. As AHL binds to the receptor TraR and subsequently activates the expressions of the *lacZ* reporter gene (McLean et al., 1997), we then quantified the production of AHL secreted by *A. tumefaciens*. Results indicated that AHPE exposure resulted in remarkable repression on 3-oxo-C8-HSL secretion. We subsequently used qRT-PCR to determine the transcriptional levels of QS-correlated genes. The expressions of *traI* and *traR* were significantly repressed compared with the untreated control. The results mentioned above combined with the docking analysis demonstrated that the breakdown of QS might be obtained by inhibiting the transcriptional levels of *traI* and *traR* rather than signal mimicry.

Previous study has shown that the virulence factors of *A. tumefaciens* are encoded by the conjugative Ti plasmid, the dissemination of which is controlled by a QS signaling based on the synthesis and perception of 3-oxo-C8-HSL (Lang and Faure, 2014). The suppression of 3-oxo-C8-HSL production would inevitably result in the reduced virulence in *A. tumefaciens*. Results from the present study showed that the swimming motility, chemotaxis, and flagella formation were significantly inhibited after exposure to AHPE. The results were in line with the decreased AHL level and repressed gene expressions, which further confirmed the dysfunctional QS of *A. tumefaciens*. It has been proven that the AHL signal 3-oxo-C8-HSL can bind to the TraR protein and the formed TraR-AHL then activates genes controlling Ti plasmid replication and conjugal transfer (Chen et al., 2004). The suppression of TraR and 3-oxo-C8-HSL secretion with AHPE treatment would result in the inhibition on Ti plasmid replication and conjugal transfer, and consequently lead to the weakened pathogenicity of *A. tumefaciens*. This has been evidence by the reduced crown gall disease after inoculation with *A. tumefaciens*. The pathogenicity of *A. tumefaciens* might be also attenuated by the inhibited motility and flagella formation. Additionally, the mediation of plasmid copy number is a complex process that might be influenced by different physiological states (Cho and Winans, 2005; Pappas, 2008; Lang and Faure, 2014).

^1^H NMR-based analysis demonstrated that the metabolism of *A. tumefaciens* was prominently disturbed with AHPE exposure. Among these changed metabolites, some were correlated with oxidative stress, quorum sensing, and protein synthesis including putrescine, arginine, isoleucine, leucine, valine, choline, and dimethylamine. Putrescine can be synthesized from arginine via arginine decarboxylase (Nakada and Itoh, 2003). The decreased arginine would inevitably result in reduced level of putrescine. It has been proven that putrescine production was significantly enhanced after supplementation with QS signals (Zhu et al., 2016). The decreased putrescine was in accordance with the decreased 3-oxo-C8-HSL level. Isoleucine, leucine, and valine are branched-chain amino acids which play important roles in maintaining cell physiological function (Zhou et al., 2019). The significant reduction of these metabolites indicated that the protein synthesis might be disordered due to the breakdown of QS in *A. tumefaciens*. Choline is one of the components of phospholipids and is involved in maintaining membrane integrity (Zhou et al., 2019). The decreased choline indicated the increased oxidative stress after AHPE treatment. To rebalance the redox level inside the cell and repair the damaged membrane caused by oxidative stress, the choline was heavily consumed. The decomposition of choline resulted in the enhanced level of dimethylamine as dimethylamine was the downstream metabolite of choline and used as carbon and nitrogen sources for cell growth (Dumas et al., 2006). The increased dimethylamine implied the disorder of protein synthesis and energy supply caused by oxidative stress.

Glutamate is crucial for glutathione (GSH) synthesis and GSH is an important redox substance for maintaining the normal reduction state of cells. The increased glutamate level indicated the intensified oxidative stress caused by AHPE. To counteract oxidative stress and reduce cell damage, more glutamate needs to be produced for GSH synthesis. In addition, betaine, one important antioxidant in organism, is essential for eliminate free radicals and maintaining membrane integrity (Schmidley, 1990). The decrease of betaine indicated the increase of free radicals. To repair the damage caused by free radicals and maintain the normal function of cells, betaine would be consumed in large quantities. In addition, the transcriptional level of *sodB* encoding SOD was significantly repressed. The decreased activity of SOD would further aggravate the oxidative stress, resulting in the disorder of physiological function of *A. tumefaciens*.

AHPE also induced disorder of energy metabolism in *A. tumefaciens*, which was evidenced by the marked increase of succinate. Succinate is an important intermediate of the tricarboxylic acid (TCA) cycle (Chen et al., 2017). The increase of succinate would inevitably result in the disorder of TCA cycle. As the most important energy source, the disorder of TCA cycle would inevitably lead to the disturbance of energy supply and eventually change the physiological function of cells. Because the energy supply was disturbed, other pathways such as anaerobic respiration would be enhanced for compensation, which could be confirmed by the significant increase in acetate. This result was in line with the research by Ringø (Ringo et al., 1984). It has been proven that the acceleration of anaerobic respiration could result in the reduced pathogenicity in organism (Lee et al., 2011). Such an effect was also confirmed by the motility assay and tobacco infection assay in this study.

In comparison with the untreated control, the levels of adenosine and AMP were notably increased. Adenosine was transformed from AMP by dephosphorylation, while AMP was derived from cyclic AMP (cAMP) by cAMP-specific phosphodiesterase and cyclic 3′,5′-phosphodiesterase (Zhou et al., 2019). The increase of AMP may be attributed to the excessive decomposition of cAMP. NADP^+^ was involved in redox reaction, reductants synthesis, and energy supply (Ying, 2008). The decrease of NADP^+^ indicated that the oxidative stress was enhanced. To counteract the oxidative stress, NADP^+^ had to be overconsumed.

In conclusion, AHPE treatment resulted in the dysfunction of QS in *A. tumefaciens* most likely by repressing the transcriptional levels of *traI* and *traR*. AHPE exposure also inhibited swimming motility, chemotaxis, and flagella formation, and lead to enhanced oxidative stress. The enhanced oxidative stress resulted in the disorder of energy metabolism, amino acid metabolism, and nuclear acid metabolism, and ultimately weakened the virulence of *A. tumefaciens*. Therefore, AHPE may act as a potential pesticide in preventing *A. tumefaciens* from infecting tobacco plants or other plant species at seedling stage.

## Data Availability Statement

The original contributions presented in the study are included in the article/Supplementary Material, further inquiries can be directed to the corresponding authors.

## Author Contributions

J-WZ, A-QJ, X-JT, and E-QL conceived and designed the experiments. J-WZ, HC, and BS performed the experiments. J-WZ, T-ZH, YH, and P-LL analyzed the data. J-WZ, A-QJ, X-JT, and E-QL wrote the manuscript. All authors contributed to the article and approved the submitted version.

## Conflict of Interest

The authors declare that the research was conducted in the absence of any commercial or financial relationships that could be construed as a potential conflict of interest.

## Footnotes

1 https://projects.biotec.tu-dresden.de/plip-web/plip/index

## Supplementary Material

Supplementary Figure 1 Chemical structure of AHPE.

Supplementary Table 1 PCR Primers for qRT-PCR.
