# Supplementary material for: 1-(4-Amino-2-Hydroxyphenyl)Ethenone Suppresses Agrobacterium tumefaciens Virulence and Metabolism
Source: Front Microbiol. 2020 Nov 12;11:584767. doi: 10.3389/fmicb.2020.584767 (PMC7688917; doi:10.3389/fmicb.2020.584767)
Supplement: Supplementary Table 1 — PCR Primers for qRT-PCR. [file Table_1.DOCX]

**Supplementary Table 1.** PCR primers for qRT-PCR.

| Genes | Primer direction | Sequence (5’-3’) | Amplicon size (bp) |
| --- | --- | --- | --- |
| *traR* | Forward | TATCCAGCACAGGCACATCA | 196 |
|  | Reverse | GGATGCGTGGTCATAGAAGG |  |
| *traI* | Forward | ATGGGATGTCGCAATAGAGG | 128 |
|  | Reverse | GTCCTGATGGCGCAAGAA |  |
| *virA* | Forward | ACCCTTCTTTACGACACGAGC | 163 |
|  | Reverse | TACGGGTTCCTTAGAAGACGG |  |
| *virG* | Forward | TAATCTCCTGGTTGCTTTCCTG | 201 |
|  | Reverse | TCAAAGAAATAGCCAGCACCT |  |
| *sodB* | Forward | TGGGAACACTCCTATTACATCG | 89 |
|  | Reverse | ACGTAGTCCCAGTTGATGAGGT |  |
| *lepA* | Forward | AGTATCCCGCTCGCTTTCA | 124 |
|  | Reverse | CGTGACCAGCTCGTGATTATT |  |
